# Cost-effectiveness analysis of tranexamic acid for the treatment of traumatic brain injury, based on the results of the CRASH-3 randomised trial: a decision modelling approach

**DOI:** 10.1136/bmjgh-2020-002716

**Published:** 2020-09-02

**Authors:** Jack Williams, Ian Roberts, Haleema Shakur-Still, Fiona E Lecky, Rizwana Chaudhri, Alec Miners

**Affiliations:** 1 Health Services Research and Policy, London School of Hygiene & Tropical Medicine, London, UK; 2 Clinical Trials Unit, London School of Hygiene & Tropical Medicine, London, UK; 3 Centre for Urgent and Emergency Care Research, School of Health and Related Research, The University of Sheffield, Sheffield, UK; 4 Emergency Department, Salford Royal Hospital NHS Foundation Trust, Salford, Salford, UK; 5 Holy Family Hospital, Rawalpindi Medical University, Rawalpindi, Pakistan

**Keywords:** health economics, public health, injury

## Abstract

**Introduction:**

An estimated 69 million traumatic brain injuries (TBI) occur each year worldwide, with most in low-income and middle-income countries. The CRASH-3 randomised trial found that intravenous administration of tranexamic acid within 3 hours of injury reduces head injury deaths in patients sustaining a mild or moderate TBI. We examined the cost-effectiveness of tranexamic acid treatment for TBI.

**Methods:**

A Markov decision model was developed to assess the cost-effectiveness of treatment with and without tranexamic acid, in addition to current practice. We modelled the decision in the UK and Pakistan from a health service perspective, over a lifetime time horizon. We used data from the CRASH-3 trial for the risk of death during the trial period (28 days) and patient quality of life, and data from the literature to estimate costs and long-term outcomes post-TBI. We present outcomes as quality-adjusted life years (QALYs) and 2018 costs in pounds for the UK, and US dollars for Pakistan. Incremental cost-effectiveness ratios (ICER) per QALY gained were estimated, and compared with country specific cost-effective thresholds. Deterministic and probabilistic sensitivity analyses were also performed.

**Results:**

Tranexamic acid was highly cost-effective for patients with mild TBI and intracranial bleeding or patients with moderate TBI, at £4288 per QALY in the UK, and US$24 per QALY in Pakistan. Tranexamic acid was 99% and 98% cost-effective at the cost-effectiveness thresholds for the UK and Pakistan, respectively, and remained cost-effective across all deterministic sensitivity analyses. Tranexamic acid was even more cost-effective with earlier treatment administration. The cost-effectiveness for those with severe TBI was uncertain.

**Conclusion:**

Early administration of tranexamic acid is highly cost-effective for patients with mild or moderate TBI in the UK and Pakistan, relative to the cost-effectiveness thresholds used. The estimated ICERs suggest treatment is likely to be cost-effective across all income settings globally.

**Key question**

**What is already known?**

- Tranexamic acid is cost-effective in reducing bleeding deaths in trauma and postpartum haemorrhage, but there is no evidence of its cost-effectiveness for those suffering traumatic brain injury (TBI).
- Evidence from the CRASH-3 randomised trial showed that the early administration of tranexamic acid reduces head injury-related deaths in patients sustaining a mild TBI with intracranial bleeding or a moderate TBI.

**What are the new findings?**

- Compared with the assumed country-specific cost-effectiveness thresholds, tranexamic acid is highly cost-effective in treating patients with moderate TBI or mild TBI with intracranial bleeding, in both the UK and Pakistan.
- Treatment was highly likely to cost-effective in probabilistic sensitivity analyses in both settings, and remained cost-effective across all deterministic sensitivity analyses.

**What do the new findings imply?**

- Providing tranexamic acid for mild and moderate TBI is likely to be cost-effective across all income settings, with the potential to prevent many thousands of premature deaths globally each year.

## Introduction

It is estimated that 69 million traumatic brain injuries (TBIs) occur each year worldwide, resulting mainly from road traffic crashes and falls.^1^ The incidence of TBI is increasing, and this trend is expected to continue as the global population ages, and as motor vehicle use increases.^2^ While the incidence of TBI is highest among high-income countries, the burden is greatest in low-income and middle-income countries (LMICs), which accounts for almost three-quarters of all TBIs.^1^ TBI can result in long-term disability or death, and can have a substantial impact on the individual’s family due to loss of income and informal caregiving. It also impacts on the economy due to healthcare expenditure and productivity loss.^2^ Around 8% of all TBIs are classified as severe (Glasgow Coma Scale (GCS) score of 3–8), which are more likely to result in death or long-lasting disability, compared with mild (GCS of 13–15) or moderate (GCS 9–12) head injury, for which the risk of death is lower, but still considerable.^1 3^

Tranexamic acid reduces bleeding by inhibiting the breakdown of blood clots. Large randomised controlled trials (RCTs) have shown its effectiveness and cost-effectiveness in reducing deaths from traumatic and postpartum haemorrhage.^4–7^ More recently, the multinational CRASH-3 trial assessed the impact of tranexamic acid on head injury deaths when administered within 3 hours of TBI, and showed a reduction in head injury deaths for those sustaining a mild or moderate head injury.^8^ However, its cost-effectiveness for this indication is yet to be assessed.

Since healthcare resources are constrained, it is important to ensure that any policy decision to fund tranexamic acid treatment in this indication results in overall net health gains. This will only occur if tranexamic acid provision replaces a less cost-effective alternative that is currently provided. For this reason, as prespecified in the trial protocol, we present an economic evaluation of tranexamic acid following TBI using the results from the CRASH-3 trial, combined with a decision modelling approach to estimate the long-term costs and benefits of treatment.^9^

## Methods

### Analysis

The economic analysis assessed the cost-effectiveness of treating patients sustaining a TBI with tranexamic acid, versus no tranexamic acid treatment, in addition to the current standard of care. The CRASH-3 trial included head injury patients (without significant extracranial bleeding) treated within 3 hours of their injury, with either a GCS score of 12 or lower, or with GCS 13–15 and any intracranial bleeding on their CT scan.^8^ Patients in the trial received a loading dose of 1 g of tranexamic acid infused over a 10 min period immediately after randomisation, followed by an intravenous infusion of 1 g over 8 hours.^8^

The trial found that tranexamic acid reduced head injury deaths among those with TBI, with a risk ratio of 0.94 (95% CI 0.86 to 1.02).^8^ However, there was evidence that people with mild TBI and intracranial bleeding (GCS score 13–15), or moderate TBI (GCS score of 9–12) had a greater benefit from tranexamic acid treatment, in terms of a reduction in head injury death (risk ratio (RR) 0.78, 95% CI 0.64 to 0.95) compared with those with a severe head injury (GCS score of 3–8, RR 0.99, 95% CI 0.91 to 1.07). For this reason, the mild and moderate CRASH-3 population was used as the base case population, excluding those with severe head injury.

We also evaluated the cost-effectiveness of tranexamic acid for alternative populations of patients, as sensitivity analyses. First, we considered people sustaining a TBI of any severity with both pupils reactive (RR 0.87 95% CI 0.77 to 0.98), while excluding those with either pupil unreactive (RR 1.03 95% CI 0.94 to 1.13). We also considered the cost-effectiveness of tranexamic acid in those sustaining a severe TBI, as there is some evidence to suggest that those in high-income countries benefit from treatment with tranexamic acid (RR 0.9, 95% CI 0.74 to 1.08), particularly when excluding severe patients with a GCS score of 3 or bilateral unreactive pupils (RR 0.62, 95% CI 0.41 to 0.96). There is no evidence of a benefit to patients sustaining a severe TBI in LMICs in either of these subgroups (see online supplementary materials).

10.1136/bmjgh-2020-002716.supp1 Supplementary data

The model evaluates the cost-effectiveness of tranexamic acid from a health service perspective in the UK and Pakistan, the two largest recruiting countries in the CRASH-3 trial (23% and 41%, respectively). This also allowed for cost-effectiveness to be assessed in a high-income and a lower-middle-income country.

The model was analysed over a lifetime time horizon with costs presented in 2018 pounds for the UK, and US dollars for Pakistan. Outcomes are presented as life-years, and quality-adjusted life years (QALYs). The model estimates the incremental cost-effectiveness ratio (ICER) by dividing the incremental costs by the incremental health outcomes, for patients receiving tranexamic acid compared with those not receiving tranexamic acid, to give a cost per life year or QALY gained. The mean age of individuals entering the model was 42 years old, as derived from the CRASH-3 trial. Both costs and outcomes were discounted equally, at a rate of 3.5% for the UK, according to National Institute for Health and Care Excellence (NICE) guidance, and a rate of 3% for Pakistan according to the International Decision Support Initiative Economic Evaluation Reference Case.^10 11^ Alternative discount rates (0% and 6%) were used in sensitivity analyses. The cost-effectiveness model was developed in Microsoft Excel, with the analysis of trial data performed in STATA V.16.

### Model structure

**Figure 1:**
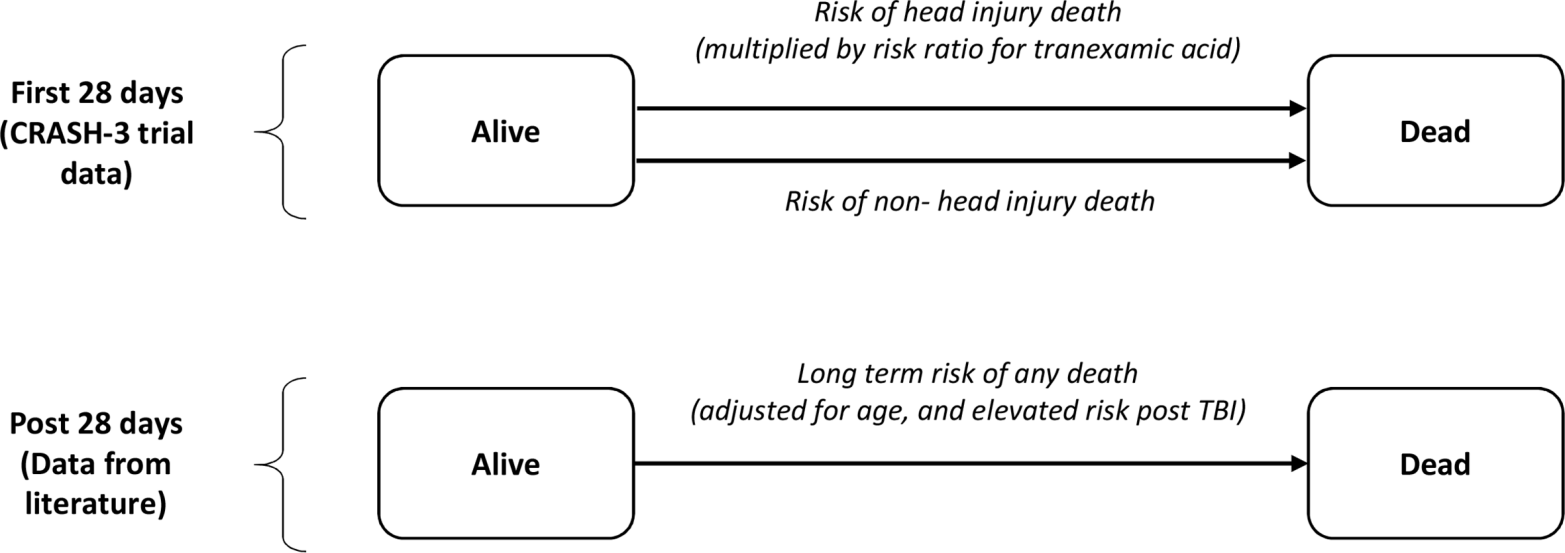
Economic model structure. TBI, traumatic brain injury.

A Markov model with time-variant transition probabilities captured the long-term outcomes associated with head injury, and is shown in figure 1. It consists of two health states, alive and dead, and includes the risk of death during the first 28 days of the trial from both head injuries and non-head injuries along with estimates of longer-term mortality. The model uses a daily cycle length for the first year, to allow the events during the trial period to be accurately modelled, followed by an annual cycle length thereafter.

### Model parameters

#### Clinical outcomes

The 28-day risk of head injury and non-head injury death for the placebo group were derived from the CRASH-3 trial, with the risk in high-income countries used to estimate the risk in the UK, and LMICs used to estimate the risk in Pakistan (table 1).^8^ A risk ratio of head injury death was applied for patients receiving tranexamic acid, as derived directly from the CRASH-3 trial. The risk of non-head injury death was equal for placebo and tranexamic acid groups.

**Table 1:** Base-case model parameters

Parameter	Value	Distribution	Source
Tranexamic acid risk ratio treatment effect			
Head injury	0.78	Log normal (μ=−0.248, σ=0.1)	CRASH-3 trial^8^
Non-head injury	1	N/A	CRASH-3 trial^8^
28 risk of death			
Head injury death (UK)	0.061	Beta (α=42, β=643)	CRASH-3 trial^8^
Head injury death (Pakistan)	0.079	Beta (α=165, β=1919)	CRASH-3 trial^8^
Non-head injury death (UK)	0.018	Beta (α=12, β=673)	CRASH-3 trial^8^
Non-head injury death (Pakistan)	0.005	Beta (α=11, β=2073)	CRASH-3 trial^8^
Long-term standardised mortality ratios			
First year, postinjury	4.00	Normal (95% CI 3.27 to 4.90)	McMillan^12^
Beyond first year, postinjury	2.26	Normal (95% CI 1.84 to 2.77)	McMillan^12^
Utility			
Good recovery	0.894	Beta (α=50, β=5.9)	Ward Fuller^15^
Moderate disability	0.675	Beta (α=30.5, β=14.7)	Ward Fuller^15^
Severe disability	0.382	Beta (α=10.9, β=17.7)	Ward Fuller^15^
Vegetative state	−0.178	Beta (α=16.1, β=−106.3)	Ward Fuller^15^
Average utility (weighted average of above disabilities)*	0.75	By component (based on above distributions for disabilities)ˆ	Calculated
Costs (UK)			
Tranexamic acid (full dose)	£6.00	N/A	BNF^18^
Sodium chloride	£3.25	N/A	BNF^18^
Needle and syringe	£0.05	N/A	NICE^20^
Hospital cost	£4741	Gamma (k=31.4, θ=0.43)†	NHS Reference costs^23^/CRASH-3 trial^8^
Monitoring costs (first year post, injury)	£11 662	By component (see online supplementary materials)	Beecham *et al* 25 / Lecky *et al* 26
Monitoring costs (after first year, postinjury)	£2505	By component (see online supplementary materials)	Lecky *et al* 26/expert opinion
Costs (Pakistan)			
Tranexamic acid (full dose)	US$1.92	N/A	Drug information system^19^
Sodium chloride	US$0.46	N/A	Drug information system^19^
Needle and syringe	US$0.20	N/A	Dziekan *et al* 21
Hospital cost	US$92	Gamma (k=21.8, θ=0.34)†	WHO CHOICE^24^/CRASH-3 trial^8^
Monitoring costs	US$0	N/A	Expert opinion

*Deterministic and probabilistic average utility values are estimated from a weighted average of CRASH-3 patients with good recovery (3094), moderate disability (1288), severe disability (677) and vegetative state (124).

†Gamma distribution for hospital length of stay (UK: 13.7 days, Pakistan: 7.4 days).

BNF, British National Formulary; N/A, not available; NHS, National Health Service; NICE, National Institute for Health and Care Excellence.

Following the 28-day trial follow-up period, the risk of death was assumed equal for people treated with and without tranexamic acid. Standardised mortality ratios (SMRs) were used to account for the higher risk of death post-TBI compared with the general population. SMRs were derived from a study which included a variety of head injury severities, which estimated an SMR of 4 for the first year following injury, and 2.26 thereafter, compared with a group of matched community controls.^12^ These SMRs were applied relative to age based, general population mortality estimates specific to both the UK and Pakistan.^13 14^ It was assumed that this excess risk of death continued throughout the duration of the model. A sensitivity analysis that excluded this long-term risk of death was performed to assess the impact of this parameter. The long-term survival estimates produced by the model are shown in the online supplementary materials.

There was no evidence of an increase in the risk of adverse events following tranexamic acid use in the CRASH-3 trial, therefore, they were not included in the model.

#### Utility

In the CRASH-3 trial, there was little difference between the Disability Rating Scale (DRS) scores reported for those with mild and moderate TBI (tranexamic acid 3.12 (SD: 5.6) vs placebo 2.91 (5.1), with lower scores indicating less disability).

Utility values were not collected as part of the CRASH-3 trial. A systematic review and EQ-5D utility mapping study was identified, which reported utility values for patients with TBI, based on their level of disability, as defined by the Glasgow Outcome Scale (GOS).^15^ In the absence of GOS outcomes in the CRASH-3 trial, we used the DRS scores of CRASH-3 trial patients to estimate the proportion of patients in each GOS category, using a qualitative estimation involving a clinical expert. This resulted in an estimated 3094 patients with a good recovery, 1288 with moderate disability, 677 with severe disability and 124 in a vegetative state. The utility values for each GOS category were then used to estimate the mean utility of CRASH-3 patients, using a weighted average. This mapping process resulted in an estimated utility of 0.75 for the mild and moderate TBI population, using a UK value set. This utility value was also used for Pakistan, in the absence of a value set for the Pakistan population (table 1). The estimated mapping of DRS scores to GOS outcomes is shown in the supplementary materials, with clinical input used in the mapping estimation.

Due to the uncertainty around the utility estimates, a sensitivity analysis considered an alternative method to estimate GOS outcomes among CRASH-3 patients, using a study reporting both GCS scores at injury and GOS outcomes (see online supplementary materials).^3 15^ This produced a higher utility of 0.79. An alternative sensitivity analysis considered a lower utility value of 0.63 (in both treatment groups), based on the midpoint of mild and moderate TBI utility values, as reported in a Swiss quality of life study.^16^ This was used to assess the impact of a lower utility estimate on the cost-effectiveness.

It was assumed that individuals who died within the 28-day study period had a utility of 0 between their injury and death. Mean utility values were adjusted by age, based on UK general population utility estimates, to account for declining utility with age.^17^

#### Costs

The model captured the costs associated with providing treatment, including the cost of tranexamic acid, needle and syringe, and nurse administration time, which were applied to the tranexamic acid arm only (table 1). The total cost of tranexamic acid (2 g total dose) was derived from the British National Formulary (£6 per person) for the UK, and from local online drug systems for Pakistan (US$1.92 per person).^18 19^ The cost of a 100 mL and a 500 mL sodium chloride infusion bag was derived from the same sources for both settings (£3.25 and US$0.46, in total).^18 19^ The costs of needles and syringes were derived from an NICE costing template for the UK, and from a global study for Pakistan.^20 21^

In the CRASH-3 trial, tranexamic acid was given in an emergency department setting. The nurse time to administer tranexamic acid was assumed to be 21 min (as per the CRASH-2 trial, involving the same treatment administration), with hourly nurse costs derived from UK social service costs.^7 22^ Administration of tranexamic acid in Pakistan was assumed to be performed by a postgraduate doctor, earning an estimated US$5.20/hour, with costs derived from a trial hospital in Pakistan.

Inpatient hospital costs were derived from UK National Health Service reference costs, and WHO-CHOICE costs for Pakistan.^23 24^ There was little difference in hospital length of stay for those treated with and without tranexamic acid, but it differed between high-income countries (13.7 days) and low-income countries (7.4 days). While the majority of patients were discharged home in the trial (85% in both arms), some patients were discharged to another hospital during their stay (tranexamic acid: 7.7%, placebo: 8.2%) or remained in hospital beyond 28 days (tranexamic acid: 7.3%, placebo: 6.4%), which was not captured in our calculation of length of stay. However, since these were balanced across arms, and since the length of stay was assumed the same, hospital costs did not impact on the incremental costs, and therefore, did not impact the cost-effectiveness in the base-case analysis. A sensitivity analysis was performed to assess the impact of this assumption, by modelling the differences in mean length of stay between treatment arms for the UK (tranexamic acid: 14 days, placebo: 13.3 days) and Pakistan (tranexamic acid: 7.3 days, placebo: 7.4 days).

Patients were assumed to incur monitoring costs post-discharge, which included primary care visits and outpatient clinic visits, as well as community care such as formal carers and rehabilitation. For the UK, first year monitoring costs were derived from a UK costing study, for those with good recovery, moderate disability and severe disability, with an estimated cost of £11,662.^25^ Long-term monitoring costs (after the first year) were derived from a previous UK health technology assessment, with costs estimated by expert opinion.^26^ The average cost was estimated to be £2505 per year and was assumed to occur until the patient died. Due to the uncertainty in these costs, we explored the impact of excluding monitoring costs beyond the first-year postinjury, and applying monitoring costs until 5 years postinjury only, in sensitivity analyses. For Pakistan, the model assumes no routine monitoring is provided postdischarge, based on input from local trial collaborators.

Costs were inflated to 2018 prices using the UK hospitals and community service index, and gross domestic product (GDP) per capita indices derived from the World Bank for Pakistan.^22 27^

### Cost-effectiveness threshold

We used the lower bound of the £20 000 to £30 000 per QALY cost-effectiveness threshold stated by NICE for the UK.^10^ The cost-effectiveness threshold in Pakistan was derived from a study of thresholds for LMICs and inflated to 2018 values.^28^ This gave an estimated threshold of US$158 per QALY threshold (estimated as 10.75% of GDP per capita). Our results assume that tranexamic acid was cost-effective when the estimated ICERs fell below these cost-effectiveness thresholds.

### Sensitivity analyses

The main analysis was performed using probabilistic sensitivity analyses to simultaneously capture the uncertainty in model parameters. Distributions were assigned to each probabilistic parameter, with each sampled simultaneously across 1000 Monte Carlo simulations. We also ensured that the probability of cost-effectiveness did not increase with higher numbers of simulations. One-way deterministic sensitivity analyses were also performed to assess the sensitivity of specific parameters on the cost-effectiveness estimates, and are presented relative to the base case as a tornado diagram. The input parameters for alternative model populations, including patients with both pupils reactive and those sustaining a severe TBI, are presented in the supplementary materials.

### Patient and public involvement

Patient and public involvement (PPI) groups were involved in various stages of the CRASH-3 trial, including the trial design, consent procedure, outcome data collection, interpretation of results and the results dissemination strategy. Although the cost-effectiveness analysis did not involve explicit PPI input, the model does capture the key outcomes of the CRASH-3 trial. The PPI groups included people at risk of, or suffering, TBI and charitable organisations involved with supporting people who have suffered trauma or TBI.^29^

## Results

The costs, life years and QALYs associated with and without tranexamic acid treatment are presented in table 2. In the base-case analysis, tranexamic acid is highly cost-effective in both the UK and Pakistan for those sustaining a moderate TBI or mild TBI with intracranial bleeding, with both ICERs below their respective willingness to pay thresholds.

**Table 2:** Base-case cost-effectiveness results for mild and moderate TBI patients treated with and without tranexamic acid in the UK and Pakistan

	Costs	LYs	QALYs	ICER (LY)	ICER (QALY)	CE threshold (per QALY)	Probability CE at threshold
UK							
Placebo	£55 110	16.87	12.10				
Tranexamic acid	£55 869	17.12	12.28	£3078	£4288	£20 000	99%
Pakistan							
Placebo	US$92	14.97	10.83				
Tranexamic acid	US$97	15.26	11.04	US$17	US$24	US$158	98%

CE, cost-effectiveness; ICER, incremental cost-effectiveness ratio; LY, life-years; QALY, quality-adjusted life-years; TBI, traumatic brain injury.

**Figure 2:**
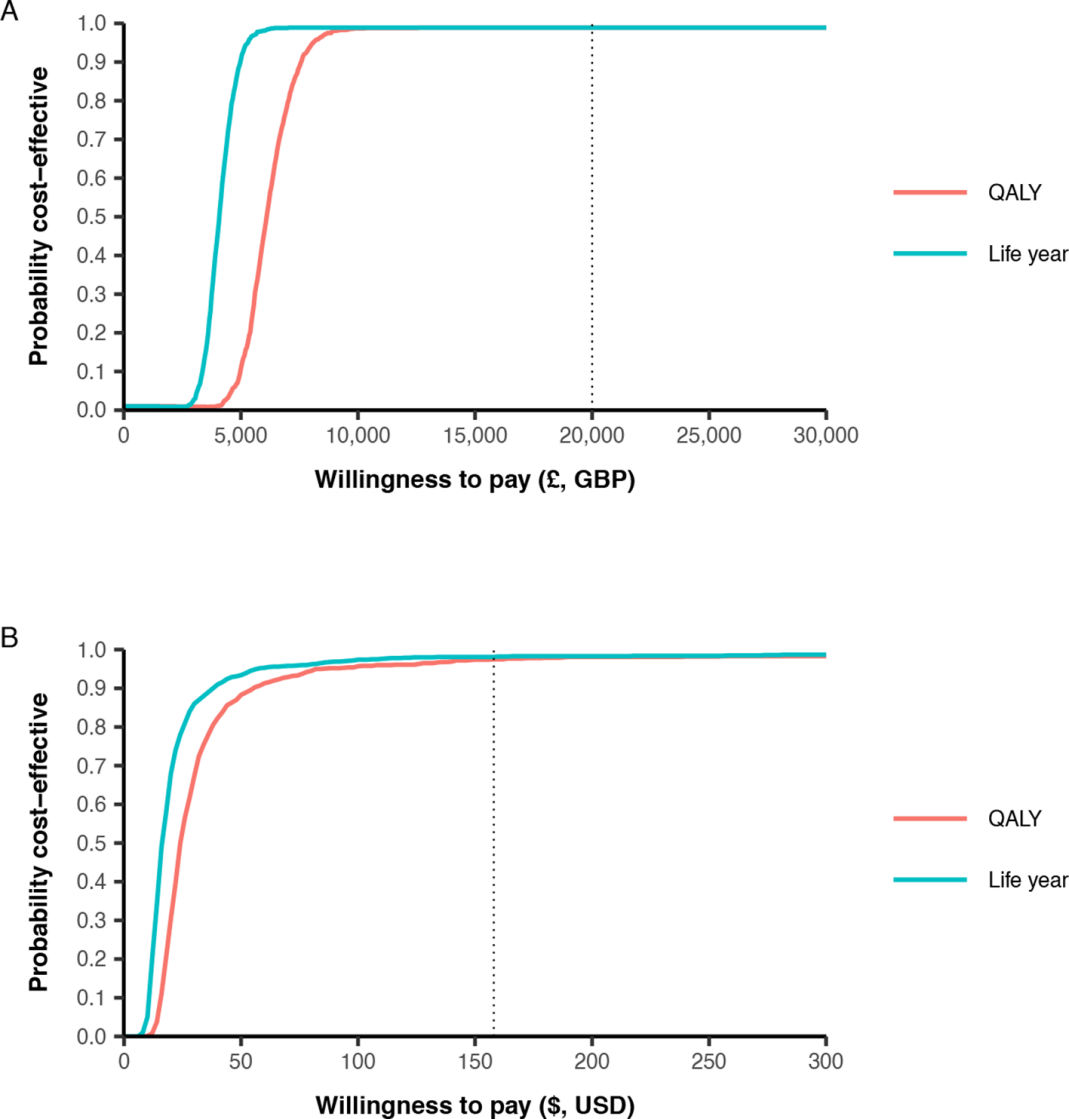
Cost-effectiveness acceptability curve for tranexamic acid for patients with mild or moderate traumatic brain injury in (A) the UK and (B) Pakistan. Dotted lines represent willingness to pay per QALY thresholds for UK (£20 000) and Pakistan (US$158). GBP, costs in pound; QALY, quality-adjusted life years; USD, US dollars.

Compared with the UK cost-effectiveness threshold of £20 000/QALY, the ICER for those with mild and moderate head injury is considerably lower at £4288/QALY, with a 99% probability of being cost-effective at this threshold (figure 2). For Pakistan, the ICER was US$24/QALY, and is again lower than the corresponding cost-effectiveness threshold of US$158/QALY, with a 98% probability to be cost-effective. When considering life years only, the ICER was £3078 per life year gained in the UK, and US$17 per life year gained in Pakistan.

There was little difference between the deterministic base-case ICERs, and the average ICER estimated across all probabilistic sensitivity analysis simulations (£4288 vs £4286 for the UK and US$24.01 vs US$24.38 for Pakistan, respectively).

For the UK, the cost of purchasing and administering tranexamic acid represented a very small proportion of the incremental costs (3%), with long-term monitoring costs contributing to most of the incremental costs for the tranexamic acid group (97%). These higher costs are due to a higher proportion of patients surviving when receiving tranexamic acid, since monitoring costs per person were equal for both treatment groups. In contrast, for Pakistan, the costs associated with tranexamic acid treatment, and treatment administration, represented all of the incremental costs between the two groups, since there were no monitoring costs. The total life years and QALYs predicted by the model were higher for the UK than Pakistan due to lower risk of death both within the trial, and beyond the trial period, for those in the UK compared with Pakistan.

### Deterministic sensitivity analysis

**Figure 3:**
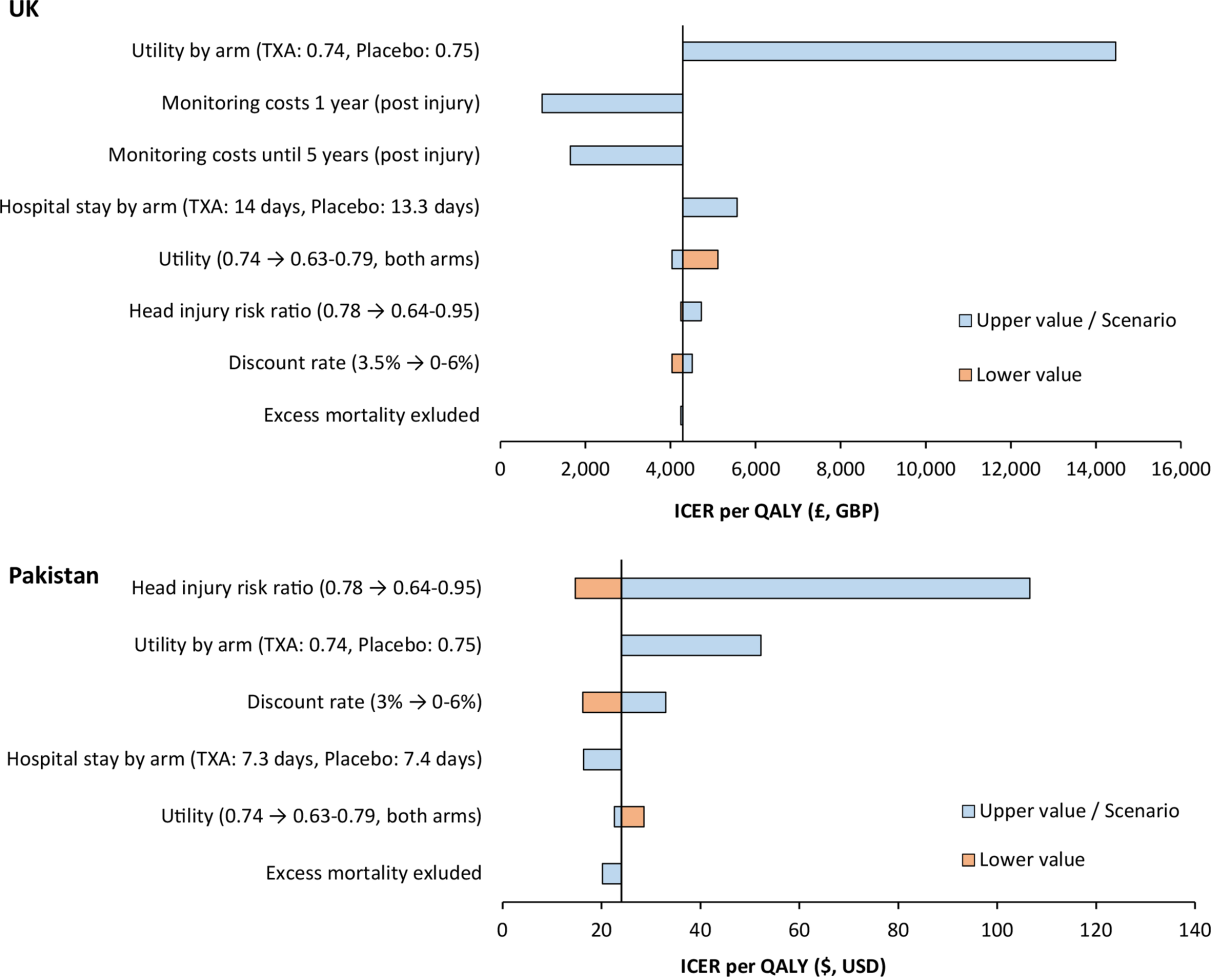
Tornado diagram showing deterministic sensitivity analyses and the impact on the ICER, in the UK and Pakistan. GBP, costs in pound; ICER, incremental cost-effectiveness ratio; QALY, quality-adjusted life years; TXA, tranexamic acid; USD, US dollar.

A number of deterministic sensitivity analyses were performed. While the estimated ICERs varied considerably by setting, tranexamic acid remained cost-effective across all sensitivity analyses in both settings, with all ICERs below the willingness to pay thresholds (figure 3).

For the UK, the largest increase in the ICER per QALY resulted when assuming a lower utility for those receiving tranexamic acid (0.74) compared with placebo (0.75) based on trial DRS scores, increasing the ICER to £14 465. Restricting monitoring costs to only the first year or first 5 years postinjury reduced the ICER to £979 and £1646, respectively. Assuming a lower utility for both groups (0.63) increased the ICER to £5112. A sensitivity analysis considering a head injury death risk ratio of 0.95 for tranexamic acid (ie, a smaller reduction in head injury deaths associated with treatment) resulted in a small increase in the ICER to £4721.

For Pakistan, the results were most sensitive to the risk ratio of head injury death associated with tranexamic acid. When increasing the risk ratio to 0.95, the ICER increased to US$107, while reducing it to 0.64 reduced the ICER to US$15. Assuming a lower utility for those receiving tranexamic acid compared with placebo increased the ICER to US$52. The remaining sensitivity analyses had minimal impact on the ICER, with none influencing the cost-effectiveness decision.

For mild and moderate TBI patients treated within 3 hours of injury, the tranexamic acid risk ratio for head injury death is lower with earlier treatment administration. A sensitivity analysis showed that earlier treatment administration, within 2 hours of injury, was associated with greater cost-effectiveness (lower ICERs) compared with our base-case results, in both settings. For example, if tranexamic was provided 30 min after injury, the estimated ICERs would fall to £4236 per QALY in the UK, and US$14 per QALY in Pakistan, based on an estimated head injury death risk ratio of 0.62. Full results, by 30 min intervals from injury to treatment, are available in the online supplementary materials.

Tranexamic acid remained cost-effective when considering a short-term time horizon of 5 years, with ICERs of £6489 and US$89 for the UK and Pakistan.

### Alternative model populations

When considering patients sustaining a TBI of any severity with both pupils reactive, tranexamic acid treatment remained highly cost-effective. The ICERs were £6097/QALY in the UK and US$24/QALY in Pakistan, and 99% and 97% likely to be cost-effective at the assumed cost-effectiveness thresholds, respectively.

For patients in high-income countries sustaining a severe TBI, there is uncertainty around the cost-effectiveness of providing tranexamic acid. The ICERs were £18 519 for all severe patients, and £18 672 in a subgroup of severe patients but excluding those with a GCS score of 3 or bilateral unreactive pupils. In probabilistic sensitivity analyses of these two groups, tranexamic acid was 62% and 65% likely to be cost-effective at the £20 000/QALY threshold, respectively. At £30 000/QALY, the upper limit of the NICE cost-effectiveness threshold, tranexamic acid was 86% and 98% likely to be cost-effective in these two groups. Full results for these analyses are available in the online supplementary materials.

## Discussion

Of the estimated 69 million TBI’s that occur globally each year, 55.9 million are mild and 7.6 million are moderate TBIs. Those with mild TBI and evidence of intracranial bleeding, and those with moderate TBI who receive tranexamic acid within 3 hours have a considerable reduction in their risk of head injury death, meaning treatment could potentially save many thousands of lives globally each year. While the cost of treatment is low, providing treatment will still incur additional costs to the health service. Our analysis shows that tranexamic acid is a highly cost-effective treatment in both high-income and lower-middle income settings for those with mild or moderate TBI, and is also cost-effective when treating patients with both pupils reactive. The results for those with mild and moderate TBI were also robust across sensitivity analyses, with tranexamic acid 99% and 98% likely to be cost-effective in the UK and Pakistan, respectively.

When putting the results of our analysis into context, tranexamic acid was cost-effective across vastly different healthcare settings. Treatment remained highly cost-effective at a relatively low cost-effectiveness threshold of 11% of GDP per capita for Pakistan, which is one of the lowest cost-effectiveness thresholds globally. In addition, the US$24 per QALY ICER for Pakistan is lower than any country’s cost-effectiveness threshold, as reported by Ochalek *et al*.^28^ It also remains considerably lower than the cost-effectiveness threshold range estimated for Pakistan (US$106–US$815) by Woods *et al*.^30^ This suggests that tranexamic acid for mild and moderate patients is likely to be cost-effective across all income settings.

In the UK, tranexamic acid was highly cost-effective, and remained cost-effective across all sensitivity analyses. NICE guidelines included tranexamic acid for the prehospital care of patients with trauma, following the results of the CRASH-2 trial.^31^ Our analysis suggests that early administration of tranexamic acid is also highly cost-effective and should be recommended for patients with moderate TBI and mild TBI with intracranial bleeding. There was considerable uncertainty in the cost-effectiveness of tranexamic acid for patients with severe TBI in the UK, although treatment was more likely to be cost-effective than not at the £20 000/QALY cost-effectiveness threshold. Treatment was highly likely to be cost-effective at cost-effectiveness threshold of £30 000/QALY.

The CRASH-3 trial showed that earlier treatment administration resulted in a greater reduction in head injury deaths for those sustaining a mild or moderate TBI. This relationship was unknown during the trial, and the randomisation process also required additional time prior to treatment. Therefore, tranexamic acid is likely to be even more cost-effective than our results suggest if the time to treatment administration is reduced in a real-world setting, as demonstrated in a sensitivity analysis. In the CRASH-3 trial, tranexamic acid was administered in the emergency department, but this finding would support its administration in the prehospital setting (eg, at the location of injury or in the ambulance), where possible.

To our knowledge, this is the first cost-effectiveness analysis of tranexamic acid for patients sustaining a TBI. Our results are similar to those of previous economic evaluations for tranexamic acid in postpartum haemorrhage, trauma patients with significant haemorrhage and patients experiencing bleeding during the surgery.^6 7 32^ These studies have also shown tranexamic acid to be cost-effective, with relatively low incremental costs associated with providing treatment.

Our analysis has considerable strengths, but also some limitations. The analysis uses data taken from a large, multinational RCT, and therefore, the evidence of the treatment effect can be considered of high quality. In addition, our analysis showed the intervention is highly likely to be cost-effective in both high-income and low-income settings. However, one limitation of our analysis is that the CRASH-3 trial only followed patients for 28 days postinjury, leading to uncertainty in patient outcomes beyond this time. Data on the long-term outcomes posthead injury are relatively scarce in the literature, particularly for LMICs. We assumed that after the trial period the risk of death remained considerably elevated compared with the general population (four times higher for first year, two times higher thereafter), to capture the long-term additional risk of death compared with the general population. These elevated risks were derived from Scotland, and therefore, may differ for low-income countries. However, sensitivity analyses using higher discount rates (giving lower weighting to future events), and a scenario excluding this additional mortality both had little impact on the estimated ICER, with tranexamic acid remaining cost-effective in both analyses.

Additionally, the CRASH-3 trial did not collect direct utility estimates, meaning that they were estimated from the DRS outcomes at 28 days (or at time of discharge). While just over half of all mild and moderate patients had no disability at discharge, there was uncertainty in the average utility of CRASH-3 trial patients, as these data were not collected in the trial. There was also uncertainty regarding the long-term disability of patients beyond 28 days. While studies have tended to show an improvement in functional outcomes up to 1-year postinjury (suggesting the utility may initially improve beyond the 28-day trial period), a long-term study over 7 years showed that functional outcomes post-TBI can improve or deteriorate over time.^33^ A limitation of our model is that we did not capture potential long-term changes in functional outcomes, instead only using outcomes reported in the trial period to capture quality of life. However, sensitivity analyses considering higher and lower utility values had little impact on the ICER in either setting, and did not change the cost-effectiveness decision.

Lastly, our analysis was performed from a health service perspective, and therefore, did not capture the potential long-term costs associated with caregiver burden or out-of-pocket medical payments for those living with disability. This may be particularly burdensome in low-income settings, where there may be more emphasis on the family providing care to individuals with disabilities. However, it should be noted that as the disability scores for survivors between the groups is similar, any additional societal burden associated with tranexamic acid treatment would only result from the higher proportion of patients surviving when receiving this treatment.

## Conclusion

We have used results from a large, multinational trial to show that the early administration of tranexamic acid is a highly cost-effective treatment for people with moderate TBI and mild TBI with intracranial bleeding, in the UK and Pakistan. Furthermore, our results suggest treatment is likely to be cost-effective across all income settings.
