# Supplementary material for: Cost-effectiveness analysis of tranexamic acid for the treatment of traumatic brain injury, based on the results of the CRASH-3 randomised trial: a decision modelling approach
Source: BMJ Glob Health. 2020 Sep 2;5(9):e002716. doi: 10.1136/bmjgh-2020-002716 (PMC7470492; doi:10.1136/bmjgh-2020-002716)
Supplement: Supplementary data [file bmjgh-2020-002716supp001.pdf]

A cost-effectiveness analysis of tranexamic acid for the treatment of traumatic brain injury, based on the results of the CRASH-3 randomised trial: A decision modelling approach

Supplementary materials

Disability Rating Scale outcomes

The disability rating scale (DRS) outcomes, stratified by population, are presented in Table 1. In order to estimate the utility and monitoring costs post TBI, we estimated the Glasgow Outcome Scale (GOS) score corresponding to each level of disability, as reported for the DRS score. This estimation process involved clinical feedback on the mapping of DRS scores to GOS outcomes.

Williams J, *et al. BMJ Global Health* 2020; 5:e002716. doi: 10.1136/bmjgh-2020-002716

Table 1: Estimating disability severity from Disability Rating Scale to estimate health state utility

DRS	Level of Disability (Based on DRS score)[1]	All patients (n)	Mild/ Moderate TBI (n)	Both pupils react (n)	Severe TBI, all patients, high income countries (n)	Severe TBI, high income countries, excluding GCS3/ unreactive pupils* (n)	Estimated Corresponding GOS outcome
0	None	3,354	2,845	3,172	167	67	Good recovery
1	Mild	336	249	306	49	18	
2-3	Partial	974	775	907	86	40	Moderate disability
4-6	Moderate	689	513	638	86	36	
7-11	Moderately Severe	633	384	539	130	56	Severe disability
12-16	Severe	301	157	245	57	27	
17-21	Extremely Severe	375	136	300	102	45	
22-24	Vegetative State	284	78	205	79	29	Vegetative state
25-29	Extreme Vegetative State	250	46	154	40	16	
**Total**		**7,196**	**5,183**	**6,466**	**796**	**334**	

DRS: Disability rating scale, GOS: Glasgow Outcome Scale. * Excluding patients with a GCS score of 3, or patients with bilateral unreactive pupils.

Utility

Utility estimation – CRASH-3 DRS scores (Base case)

Utility values were estimated from a systematic review and EQ-5D utility mapping study, providing utility values stratified by GOS outcomes.[2] Utilities were then estimated using the UK value set for EQ-5D scores (Table 2). These scores were combined with the proportion of patients falling into each outcome (Table 1), to estimate the overall utility across each of the model populations, using a weighted average (Table 3).

Table 2: Mapping of Disability Rating Scale score to utilities associated with GOS outcomes

GOS categories	Estimated equivalent DRS scores*	Utility value	Distribution
Full recovery	N/A	1	N/A
Good recovery	0-1	0.89	Beta (α=50, β=5.9)
Moderate disability	2-6	0.68	Beta (α=30.5, β=14.7)
Severe disability	7-21	0.38	Beta (α=10.9, β=17.7)
Vegetative state	22-29	-0.18	Beta (α=16.1, β=-106.3)

GOS: Glasgow Outcome Scale, DRS: Disability Rating Scale

* Estimated mapping between DRS scores and GOS outcome shown in Table 1.

Table 3: Average utility across each of the model populations, using Disability Rating Scale to estimate utility

Population	Average utility
All patients	0.67
Mild / Moderate TBI	0.75
Patients with both pupils reactive	0.70
Severe TBI, all patients, high income countries	0.50
Severe TBI, high income countries, excluding GCS3/ bilateral unreactive pupils	0.50

The post-TBI utility estimates were derived from a cohort with a median age of 50 years old.[2] To account for the reduction in utility with age, a utility decrement was applied for those reaching age 55 or over (Table 4).[3] The utility estimates were not inflated between 42 (average starting age in the model) and 44, to remain conservative.

Table 4: UK general population age-based utility values and utility decrements[3]

Age	Utility	Utility decrement
35-44	0.91	0
45-54	0.85	0
55-64	0.8	0.05
65-74	0.78	0.07
≥75	0.73	0.12

For example, the average utility for patients with mild/moderate TBI after discharge would be 0.75, until they reach the age of 55, upon which the utility would decrease to 0.70 (0.75 - 0.05). The utility would then decrease to 0.68 at age 65 (0.75 - 0.07).

We also assessed the impact of using an alternative source of general population utility estimates, using an equation published by Ara and Brazier 2010.[4] This however had very little impact upon the results, leading to marginally lower ICERs in both settings.

Utility estimation – Correlation between GCS score and GOS from previous RCT (Scenario)

An alternative estimation process was considered, to predict the utility in each population. A previous analysis showed the distribution of GOS outcomes (good recovery, moderate disability, severe disability) stratified by GCS score.[5,6]

For a sensitivity analysis, we used the GCS scores from the CRASH-3 patients to estimate a distribution of GOS scores, to which the utility values estimated by Ward Fuller *et al.* (Table 2) were applied.

Table 5: Distribution of Glasgow Outcome Scale outcomes, by Glasgow Coma Scores at injury, derived from previous CRASH trial [5,6]

Glasgow Coma Scale (GCS) at injury	Glasgow Outcome Scale (GOS) amongst survivors		
	**Good recovery**	**Moderate disability**	**Severe Disability**
3	28.9%	30.8%	40.3%
4	20.6%	25.8%	53.6%
5	22.9%	30.6%	46.5%
6	33.4%	34.0%	32.6%
7	44.0%	29.9%	26.1%
8	45.9%	32.7%	21.4%
9	56.8%	26.0%	17.2%
10	57.7%	27.1%	15.2%
11	65.2%	22.7%	12.0%
12	68.5%	19.7%	11.8%
13	75.2%	16.2%	8.6%
14	74.5%	16.6%	9.0%
15*	74.5%	16.6%	9.0%

GCS: Glasgow Coma Scale, GOS: Glasgow Outcome Scale

* GCS score of 15 assumed equal distribution of severity to GCS score of 14 in the absence of data

Table 6: Distribution of Glasgow Outcome Scale outcomes and estimated utility for CRASH-3 patients, for patients in each model population

CRASH-3 population	GOS outcome amongst survivors			Estimated Utility*
	**Good recovery**	**Moderate disability**	**Severe Disability**	
All patients	61.3%	22.6%	16.1%	0.75
Mild or Moderate TBI	59.4%	23.2%	17.4%	0.79
Both pupils reactive	68.5%	20.1%	11.4%	0.76
Severe TBI, all patients, high income countries	36.6%	30.8%	32.6%	0.64
Severe TBI, high income countries, excluding GCS3/ bilateral unreactive pupils	31.6%	30.9%	37.5%	0.66

GOS: Glasgow Outcome Scale

* Utility estimated by weighted average of GOS scores, based on utility estimates reported in Table 2

Williams J, *et al. BMJ Global Health* 2020; 5:e002716. doi: 10.1136/bmjgh-2020-002716

Monitoring costs

Monitoring costs for the first year post-TBI were derived from a UK costing study, and costs were stratified by GOS categories (Table 7).[7] Beyond the first year, annual costs were estimated by expert opinion, as part of a previous health technology assessment for patients with TBI.[8]

Table 7: Mapping of DRS score to GOS scores to estimate monitoring costs, for first year after head injury

GOS categories	Estimated equivalent DRS scores	Cost, first year (£)^	Distribution	Cost, after first year (£)^	Distribution
Good recovery	0-1	£290	Gamma (k=25, θ=9.6)	£26	Gamma (k=25, θ=0.96)
Moderate disability	2-6	£20,745	Gamma (k=25, θ=686)	£1,710	Gamma (k=25, θ=64)
Severe disability	7-21	£40,983	Gamma (k=25, θ=1,356)	£13,363	Gamma (k=25, θ=500)
Vegetative state	22-29	£40,983*	Gamma (k=25, θ=1,356)	£13,363*	Gamma (k=25, θ=500)

GOS: Glasgow Outcome Scale, DRS: Disability Rating Scale

*Assumed equal to severe disability. ^Cost inflated to 2018 prices (Inflator: 2007 = 1.21, 2012 = 1.07)

The average monitoring costs for the UK were estimated by combining the annual cost by GOS categories (Table 7), with the proportion of patients across each GOS (Table 1). A weighted average was used to provide the average annual monitoring cost for each population, as displayed in Table 8. For Pakistan, it was assumed that there was no monitoring costs post-discharge.

Table 8: Average monitoring costs, by CRASH-3 population, stratified by time since TBI

Population	Cost, 0-12 months (£)	Cost, >12 months (£)
Mild / Moderate TBI	£11,662	£2,505
Patients with both pupils reactive	£14,259	£3,405
All patients	£15,439	£3,831
Severe TBI, all patients, high income countries	£25,568	£7,226
Severe TBI, high income countries, excluding GCS3/ bilateral unreactive pupils	£26,022	£7,317

Williams J, *et al. BMJ Global Health* 2020; 5:e002716. doi: 10.1136/bmjgh-2020-002716

Long term model survival predictions

The survival of patients by treatment groups, and country, and shown for the first 3 months of the model (Figure 1), and for the duration of the model time horizon (Figure 2).

Figure 1: Model predictions for survival for 3 months, by treatment group, and country

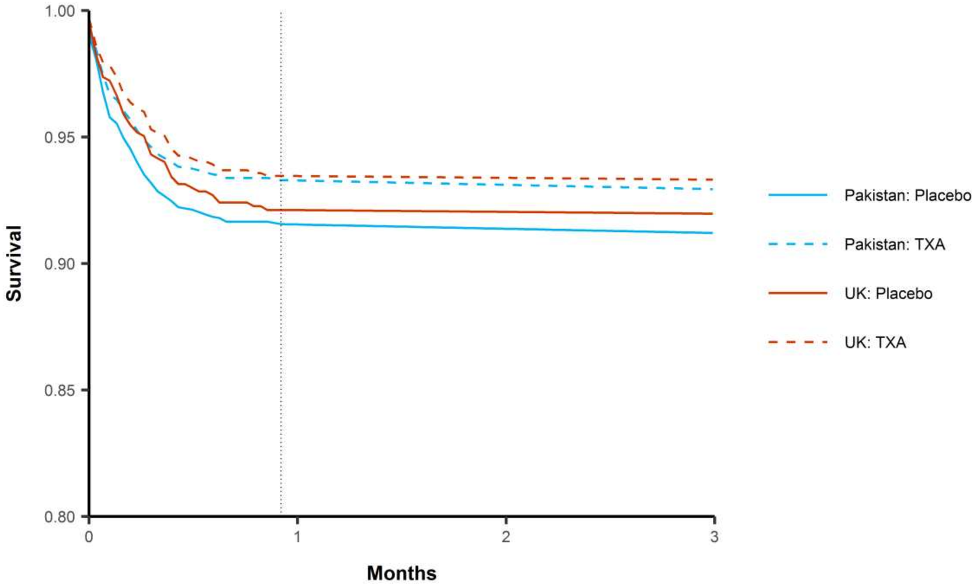

*Dotted line represents 28-day trial period. TXA: Tranexamic acid

Figure 2: Model predictions for survival for the duration of the analysis time horizon, by treatment group, and country

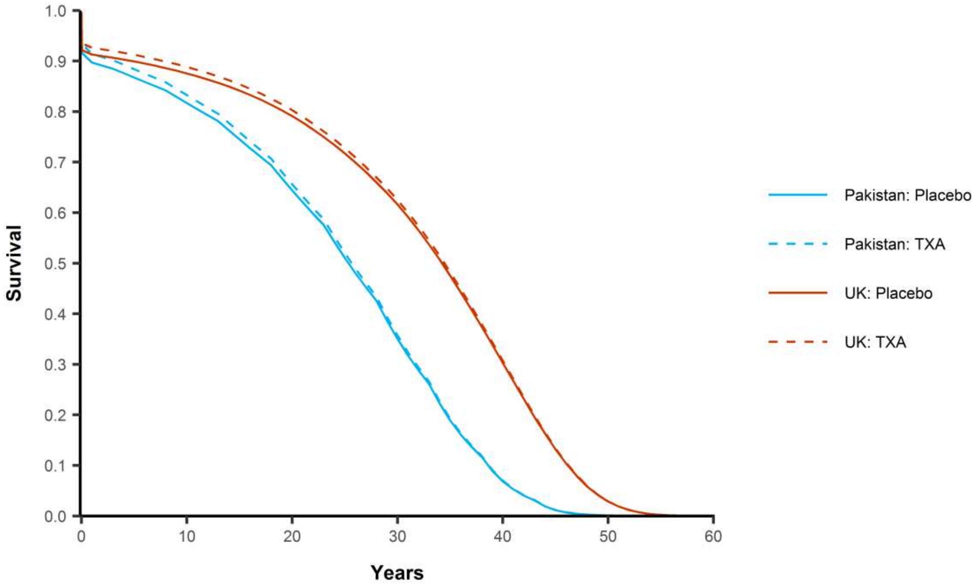

TXA: Tranexamic acid

8

Additional results: Time to treatment

We used the outputs of a clinical analysis which estimated the impact of tranexamic acid upon head injury death risk ratios in the mild and moderate TBI.[9] These statistical models adjusted for GCS, systolic blood pressure and age in a multivariable model. The estimated risk ratios and the estimated ICERs per QALY gained for each risk ratio, is presented in Table 9.

Table 9: Effect of time to treatment administration of tranexamic acid on the cost-effectiveness of tranexamic acid, in mild and moderate TBI patients

Time to treatment (mins)	Risk Ratio	ICER per QALY (UK)	ICER per QALY (Pakistan)
0	0.58	£4,229	$13
30	0.62	£4,236	$14
60	0.66	£4,244	$16
90	0.71	£4,256	$18
120	0.75	£4,274	$21
150	0.80	£4,300	$26
180	0.85	£4,349	$36

Patients sustaining TBI (any severity) with both pupil's reactive population: Data inputs and results

For the population of patients with both pupils reactive, several of the key input parameters differed compared to the mild or moderate TBI population (Table 10).

This included the background risk of head injury and non-head injury death, and risk ratio of head-injury death for patients receiving tranexamic acid. These parameters were derived directly from the CRASH-3 trial. The utility for patients with both pupils reactive, and the long term monitoring costs (for the UK only) were calculated from the distribution of DRS scores, as described in the 'Utility' and 'Monitoring costs' sections above (Tables 5-7, 9 and 10). All other parameters, which includes all costs and the standardised mortality ratios, were the same as the mild and moderate TBI population.

Table 10: Model parameters for all patients with both pupils reactive

Parameter	Value	Distribution	Source
**Tranexamic acid risk ratios**			
Head-injury	0.87	Lognormal (μ=-0.138, σ=0.06)	CRASH-3
Non-head injury	1	N/A	CRASH-3
**28 risk of death**			
Head injury death (UK)	0.105	Beta (α=109, β=930)	CRASH-3
Head injury death (Pakistan)	0.143	Beta (α=384, β=2305)	CRASH-3
Non-head injury death (UK)	0.019	Beta (α=20, β=1019)	CRASH-3
Non-head injury death (Pakistan)	0.008	Beta (α=21, β=2668)	CRASH-3
**Utility**			
Average utility	0.70	Beta, by component (see Table 2)	[2]
**Costs**			
Hospital cost (UK)	£5,158	Gamma (k=34.7, θ=0.43)*	[10] / CRASH-3
Hospital cost (Pakistan)	$102	Gamma (k=19.5, θ=0.42)*	[11] / CRASH-3
Monitoring costs (first year, UK)	£14,259	Gamma, by component (see Table 7)	[7,8]
Monitoring costs (after first year, UK)	£3,405	Gamma, by component (see Table 7)	[8]

*Gamma distribution for hospital length of stay (UK: 15 days, Pakistan: 8.1 days)

The results for the population of patients with both pupils reactive are shown in Table 11. For this population, tranexamic acid is highly cost-effective in both the UK and Pakistan, with an ICER of £6,097 in the UK, and $24 in Pakistan.

Compared to the UK cost-effectiveness threshold of £20,000/QALY, tranexamic acid is 99% likely to be cost-effective in the probabilistic sensitivity analysis (Figure 3). For Pakistan, tranexamic acid was 97% likely to be cost-effective at the estimated $158/QALY willingness to pay threshold.

When considering life years only, the ICER was £4,066 per life year gained in the UK, and $16 per life year gained in Pakistan.

Similar to the results of the deterministic results for the mild and moderate TBI population, the DSA results for patients with both pupils reactive show that for all sensitivity analyses in the UK, tranexamic acid remained cost-effective in all sensitivity analyses (Figure 4). For Pakistan, the only scenario that increased the ICER above the cost-effectiveness threshold was assuming that the risk ratio of tranexamic acid on head injury death reduced from 0.8 to 0.98, which increased the ICER to $179.

Table 11: Cost-effectiveness results for all patients with both pupils reactive

	**Costs**	**Life Years**	**QALYs**	**ICER (LY)**	**ICER (QALY)**	**CE threshold (per QALY)**	**Probability CE at threshold**
**UK**							
Placebo	£68,894	16.04	10.69				
Tranexamic acid	£69,901	16.29	10.86	£4,066	£6,097	£20,000	99%
**Pakistan**							
Placebo	$102	13.89	9.34				
Tranexamic acid	$106	14.19	9.55	$16	$24	$158	97%

ICER: Incremental cost-effectiveness ratio, LY: Life-years, QALY: Quality adjusted life-years

Williams J, *et al. BMJ Global Health* 2020; 5:e002716. doi: 10.1136/bmjgh-2020-002716

Figure 3: Cost-effectiveness acceptability curve for tranexamic acid treatment for patients with both pupils reactive, in A) the UK and B) Pakistan

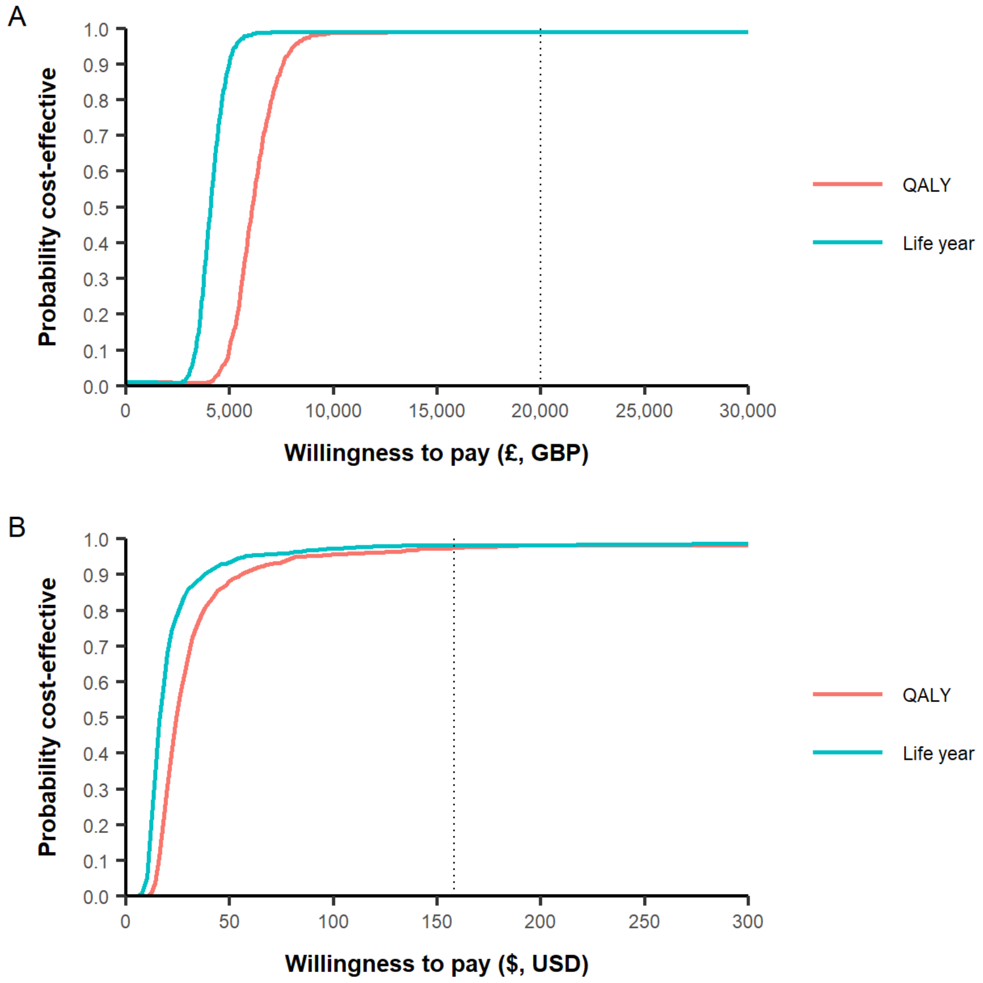

*Dotted lines represent willingness to pay per QALY thresholds for UK (£20,000) and Pakistan ($158)

Figure 4: Tornado diagram showing deterministic sensitivity analyses and the impact upon the ICER for all patients with both pupils reactive, in the UK and Pakistan

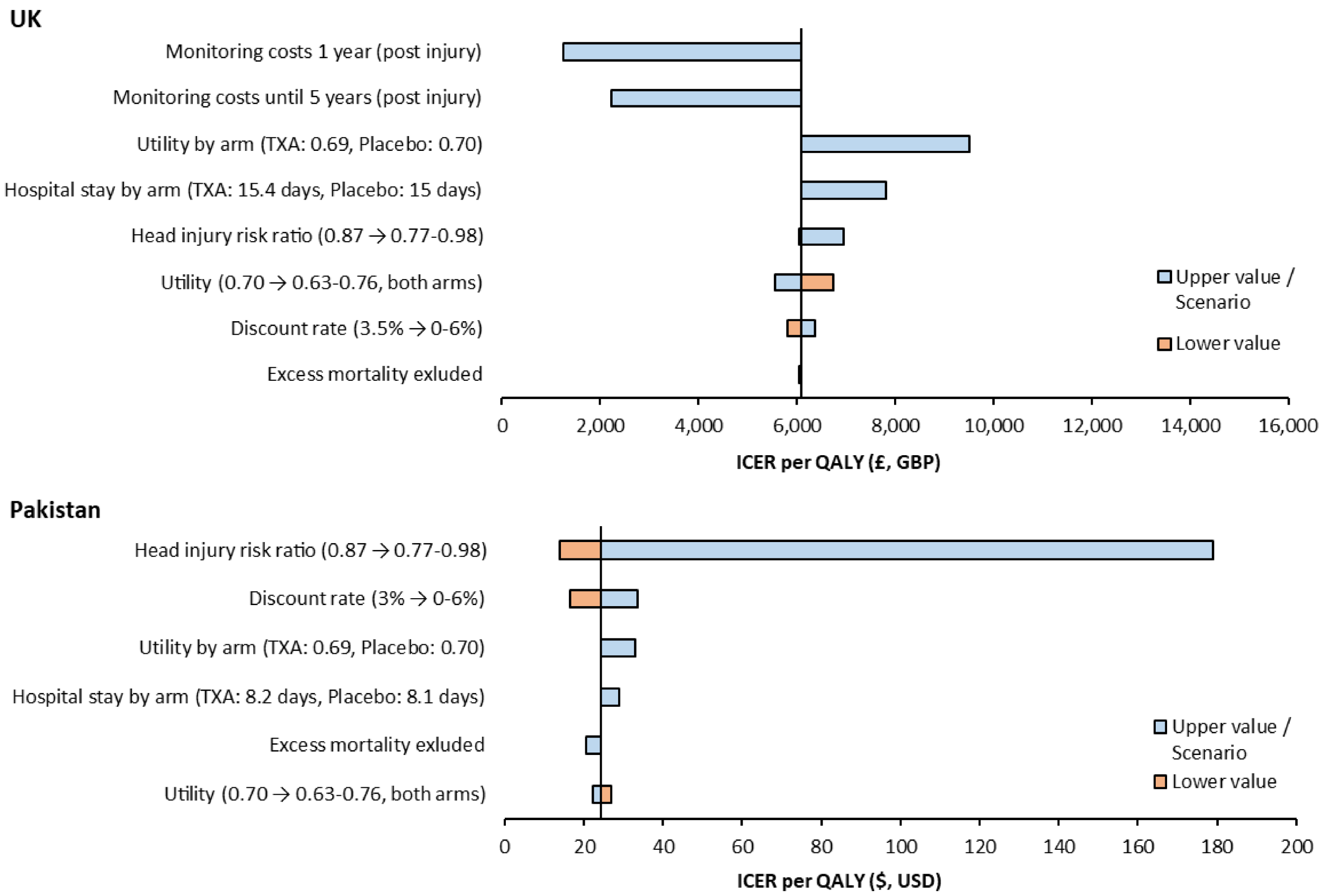

TXA: Tranexamic acid

Severe TBI, in high income countries only: Data inputs and results

For all CRASH-3 patients with a severe TBI (GCS 3-8), there was no evidence that tranexamic acid reduced head injury deaths, with a risk ratio of 0·99 (95% CI: 0·91–1·07).

However, there was some evidence that for those sustaining a severe TBI, the effect of tranexamic acid differed by income setting. In high income countries, the head injury death risk ratio is 0.9 (0.74–1.08) for those sustaining a severe TBI, whilst in low and middle income countries the risk ratio is 1.03 (0.94-1.12).

Furthermore, a subgroup analysis of patients experiencing severe TBI, but excluding those patients with a GCS score of 3 or bilateral unreactive pupils (a sensitivity analysis pre-specified in the trial), the tranexamic acid head injury deaths risk ratio is 0.62 (0.41-0.96) in high income countries, and 1.01 (0.88-1.15) in low and middle income countries.

The following analyses estimate model parameters from i) all patients experiencing severe TBI in high income countries and ii) patients experiencing severe TBI, excluding those with a GCS score of 3 or bilateral unreactive pupils. These analyses used model parameters derived from CRASH-3 data from high-income countries only. As such, the model presents the results in a UK setting only.

The two analyses of patients sustaining severe TBI were parameterised with key input data, derived directly from the CRASH-3 trial. This included the background risk of head injury and non-head injury death, and risk ratio of head-injury death for patients receiving tranexamic acid. The utility for patients with both pupils reactive, and the long term monitoring costs were calculated from the distribution of DRS scores, as described in the 'Utility' and 'Monitoring costs' sections above. The mean age was also higher (mean age of 49 for both severe TBI groups, compared to 42 for those sustaining a mild or moderate TBI, or patients with both pupils reactive). All other parameters were the same as the mild and moderate TBI population.

The SMR estimates used in the base case analysis were derived from a TBI population of mixed severity, therefore an additional sensitivity analysis for patients sustaining a severe TBI is presented, to assess the impact of higher SMR estimates upon the ICER.

Severe TBI (all patients, high income countries)

For patients sustaining a severe TBI in high income countries, the key input parameters are presented in Table 12.

Table 12: Model parameters, for all patients sustaining a severe TBI from high income countries

**Parameter**	**Value**	**Distribution**	**Source**
**Tranexamic acid risk ratios**			
Head-injury	0.9	Lognormal (μ=-0.108, σ=0.097)	CRASH-3
Non-head injury	1	N/A	CRASH-3
**28 risk of death**			
Head injury death (UK)	0.232	Beta (α=159, β=526)	CRASH-3
Non-head injury death (UK)	0.018	Beta (α=12, β=673)	CRASH-3
**Utility**			
Average utility	0.50	Beta, by component (see Table 2)	[2]
**Costs**			
Hospital cost	£5,439	Gamma (k=32.4, θ=0.49)*	[10] / CRASH-3
Monitoring costs (first year)	£25,568	Gamma, by component (see Table 7)	[7,8]
Monitoring costs (after first year)	£7,226	Gamma, by component (see Table 7)	[8]

*Gamma distribution for hospital length of stay (15.9 days)

The results for the severe TBI patients from high income countries are shown in Table 13. For this population, the base case ICER suggests treatment is cost-effectiveness for tranexamic acid, with an ICER of £18,519 in the UK. However, there is a high degree of uncertainty in these results. Compared to the UK cost-effectiveness threshold of £20,000/QALY, tranexamic acid is 61.7% likely to be cost-effective in the probabilistic sensitivity analysis (Figure 5). At the higher UK cost-effectiveness threshold of £30,000/QALY, tranexamic acid was 86.3% likely to be cost-effective. The cost-effectiveness acceptability curve presented in Figure 5 takes an unusual shape due to the correlation between the incremental costs and incremental QALY's associated with treatment, with both positive and negative incremental values for tranexamic acid compared to placebo. This correlation is shown in a cost-effectiveness plane, in Figure 6. The results of the DSA are presented in Figure 7 and Table 14.

Table 13: Cost-effectiveness results for all patients sustaining a severe TBI, for the UK

	**Costs**	**Life Years**	**QALYs**	**ICER (LY)**	**ICER (QALY)**	**CE threshold (per QALY)**	**Probability CE at threshold**
Placebo	£104,084	11.84	5.36				
tranexamic acid	£107,235	12.21	5.53	£8,400	£18,519	£20,000	61.7%

ICER: Incremental cost-effectiveness ratio, LY: Life-years, QALY: Quality adjusted life-years

Figure 5: Cost-effectiveness acceptability curve for tranexamic acid treatment for all patients sustaining a severe TBI, for the UK

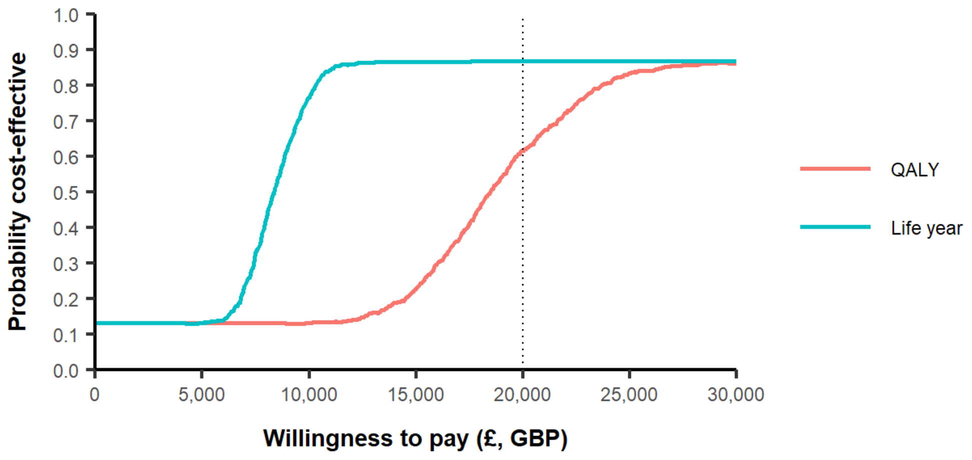

*Dotted line represents willingness to pay per QALY threshold for UK (£20,000)

Figure 6: Cost-effectiveness plane for tranexamic acid treatment for patients sustaining a severe TBI, excluding those with a GCS score of 3 and those with bilateral unreactive pupils, for the UK

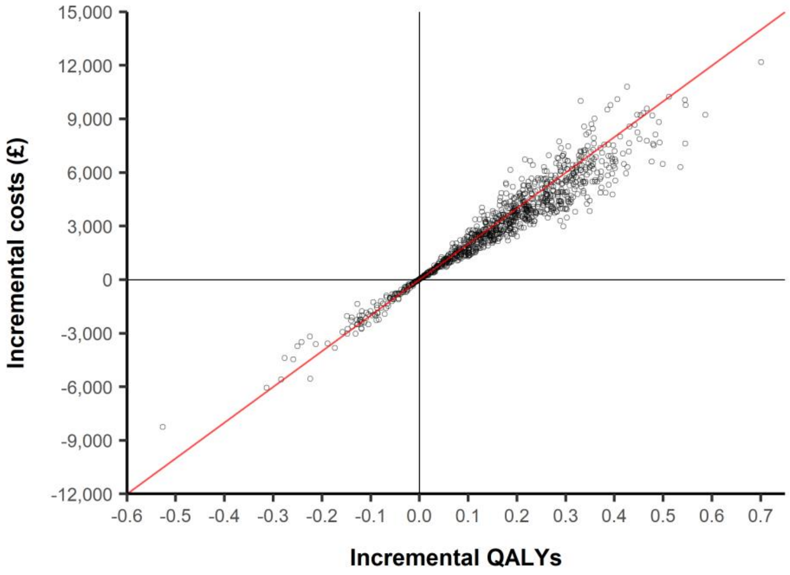

Red line represents the UK willingness to pay threshold (£20,000/QALY). Circles underneath the red line represent a cost-effective simulation.

Figure 7: Tornado diagram showing deterministic sensitivity analyses and the impact upon the ICER for all patients sustaining a severe TBI, for the UK

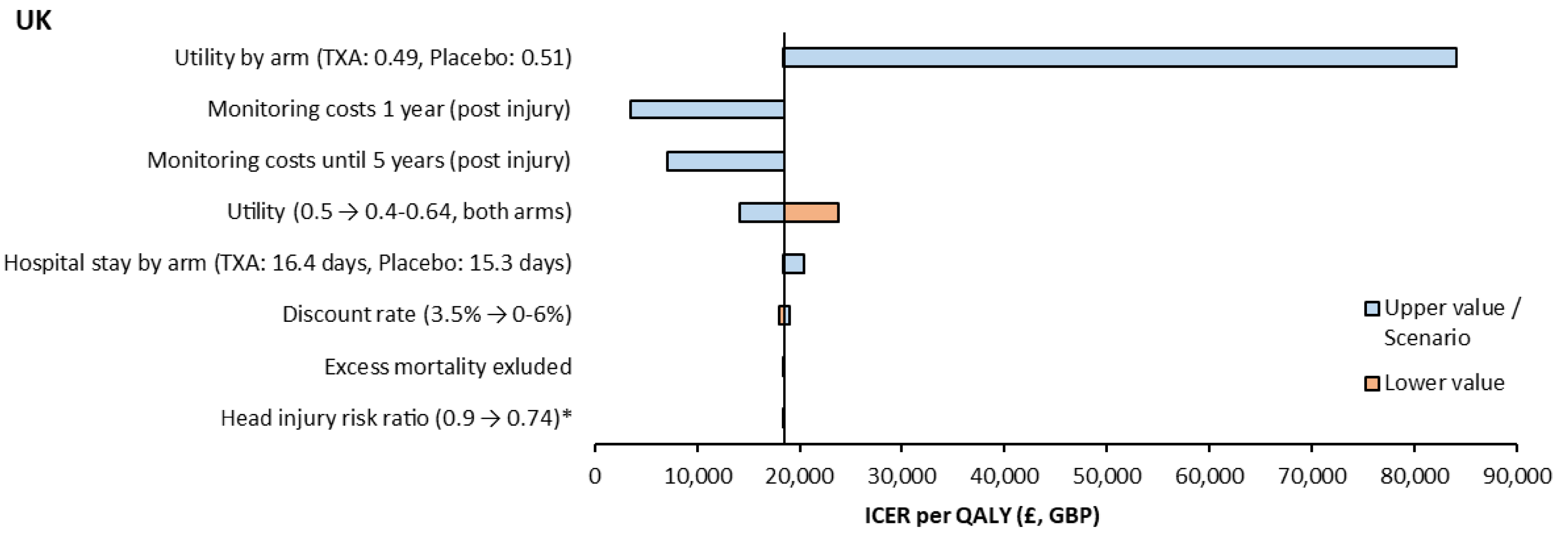

TXA: Tranexamic acid. * Risk ratio upper 95% confidence interval of 1.08 not considered.

Table 14: Deterministic sensitivity analysis of standardised mortality ratios for all patients sustaining a severe TBI, for the UK

Standardised mortality ratio*	ICER (UK)
4	£18,680
6	£18,882
8	£19,088
10	£19,295

*Standardised mortality ratio applied throughout model time horizon.

Severe TBI (excluding those with GCS score of 3 or bilateral unreactive pupils, high income countries)

For patients with severe TBI in high income countries, excluding those with GCS score of 3 and bilateral unreactive pupils, the key input parameters are presented in Table 15.

Table 15: Model parameters for patients sustaining a severe TBI, excluding those with a GCS score of 3 and those with bilateral unreactive pupils, from high income countries

**Parameter**	**Value**	**Distribution**	**Source**
**Tranexamic acid risk ratios**			
Head-injury	0.62	Lognormal (μ=-0.471, σ=0.221)	CRASH-3
Non-head injury	1	N/A	CRASH-3
**28 risk of death**			
Head injury death (UK)	0.053	Beta (α=35, β=627)	CRASH-3
Non-head injury death (UK)	0.005	Beta (α=3, β=659)	CRASH-3
**Utility**			
Average utility	0.50	Beta, by component (see Table 2)	[2]
**Costs**			
Hospital cost	£6,049	Gamma (k=45.5, θ=0.39)*	[10] / CRASH-3
Monitoring costs (first year)	£26,022	Gamma, by component (see Table 7)	[7,8]
Monitoring costs (after first year)	£7,317	Gamma, by component (see Table 7)	[8]

*Gamma distribution for hospital length of stay (17.8 days)

The results for the severe TBI patients from high income countries are shown in Table 16. For this population, the base case ICER suggests treatment is cost-effectiveness for tranexamic acid, with an ICER of £18,672, and a 64.9% probability of cost-effectiveness at the UK cost-effectiveness threshold of £20,000/QALY in the probabilistic sensitivity analysis (Figure 8). At the higher UK cost-effectiveness threshold of £30,000/QALY, tranexamic acid was 98% likely to be cost-effective. The incremental costs and incremental QALY's associated with treatment are highly correlated, with both positive and negative incremental values for tranexamic acid compared to placebo (Figure 9). When considering life years only, the ICER was £8,527 per life year gained. The results of the DSA are presented in Figure 10 and Table 17.

Table 16: Cost-effectiveness results for patients sustaining a severe TBI, excluding those with a GCS score of 3 and those with bilateral unreactive pupils, for the UK

	**Costs**	**Life Years**	**QALYs**	**ICER (LY)**	**ICER (QALY)**	**CE threshold (per QALY)**	**Probability CE at threshold**
Placebo	£131,633	14.87	6.78				
tranexamic acid	£134,302	15.18	6.93	£8,527	£18,672	£20,000	64.9%

ICER: Incremental cost-effectiveness ratio, LY: Life-years, QALY: Quality adjusted life-years

Figure 8: Cost-effectiveness acceptability curve for tranexamic acid treatment for patients sustaining a severe TBI, excluding those with a GCS score of 3 and those with bilateral unreactive pupils, for the UK

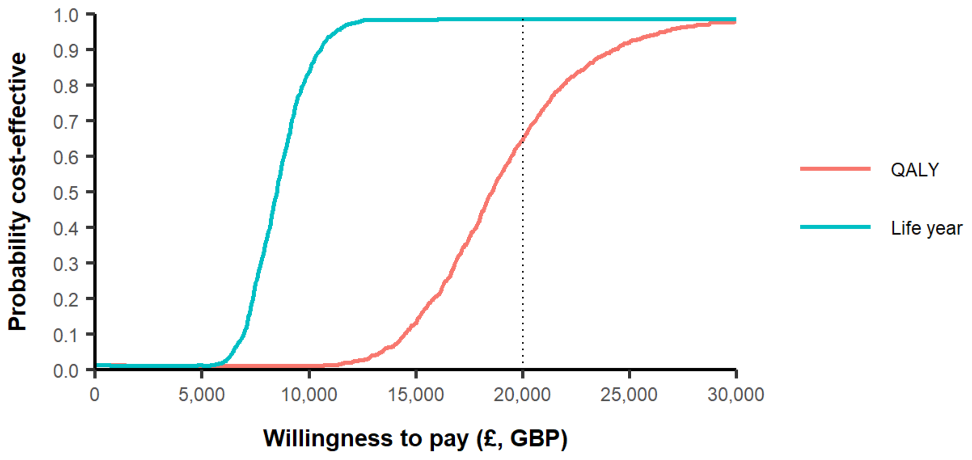

*Dotted line represents willingness to pay per QALY threshold for UK (£20,000)

Figure 9: Cost-effectiveness plane for tranexamic acid treatment for patients sustaining a severe TBI, excluding those with a GCS score of 3 and those with bilateral unreactive pupils, for the UK

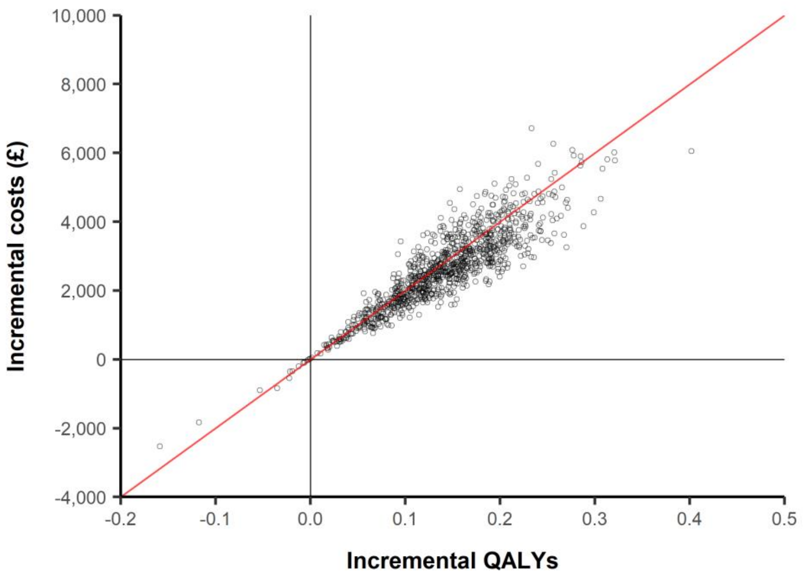

Red line represents the UK willingness to pay threshold (£20,000/QALY). Circles underneath the red line represent a cost-effective simulation.

Figure 10: Tornado diagram showing deterministic sensitivity analyses and the impact upon the ICER for patients sustaining a severe TBI, excluding those with a GCS score of 3 and those with bilateral unreactive pupils, for the UK

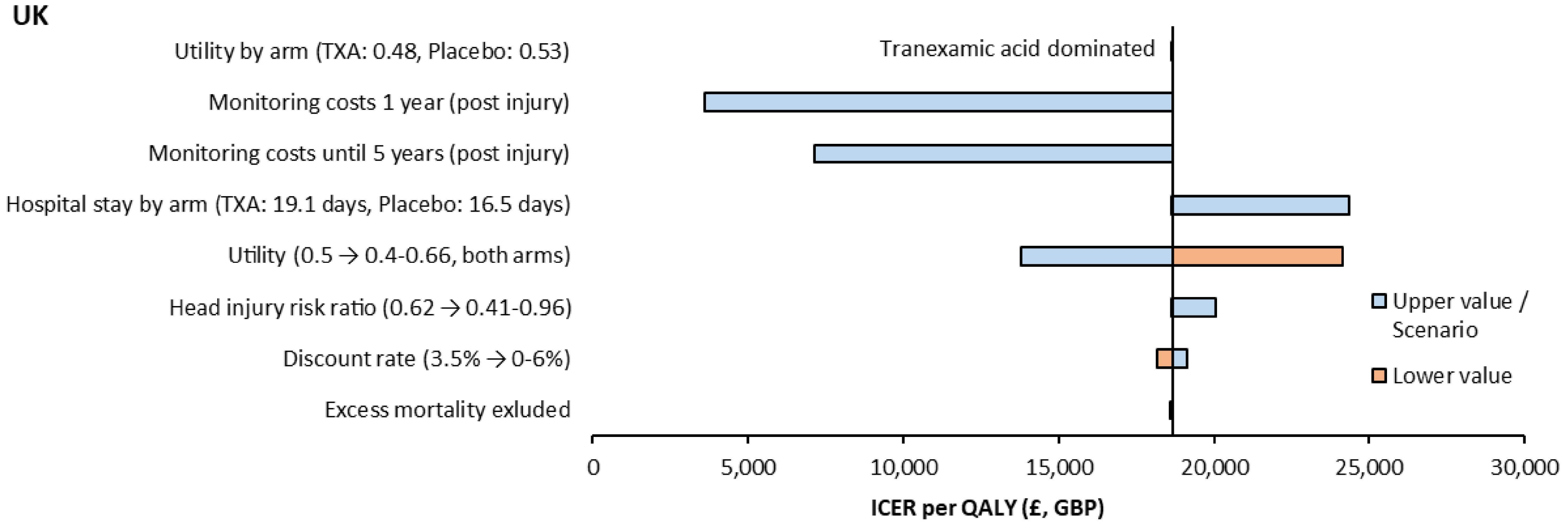

TXA: Tranexamic acid

Table 17: Deterministic sensitivity analysis of standardised mortality ratios for patients sustaining a severe TBI, excluding those with a GCS score of 3 and those with bilateral unreactive pupils, for the UK

**Standardised mortality ratio***	**ICER (UK)**
4	£18,680
6	£18,882
8	£19,088
10	£19,295

*Standardised mortality ratio applied throughout model time horizon.

References

1. Rappaport M, Hall KM, Hopkins K, Belleza T, Cope DN. Disability rating scale for severe head trauma: coma to community. Arch Phys Med Rehabil. 1982;63(3):118-23.
2. Ward Fuller G, Hernandez M, Pallot D, Lecky F, Stevenson M, Gabbe B. Health State Preference Weights for the Glasgow Outcome Scale Following Traumatic Brain Injury: A Systematic Review and Mapping Study. Value Health. 2017;20(1):141-51.
3. Kind P, Hardman G, Macran S. UK Population Norms for EQ-5D. Centre for Health Economics, University of York; 1999.
4. Ara R, Brazier JE. Populating an economic model with health state utility values: moving toward better practice. Value Health. 2010;13(5):509-18.
5. Teasdale G, Maas A, Lecky F, Manley G, Stocchetti N, Murray G. The Glasgow Coma Scale at 40 years: standing the test of time. Lancet Neurol. 2014;13(8):844-54.
6. MRC Crash Trial Collaborators, Perel P, Arango M, Clayton T, Edwards P, Komolafe E, et al. Predicting outcome after traumatic brain injury: practical prognostic models based on large cohort of international patients. BMJ. 2008;336(7641):425-9.
7. Beecham J, Perkins M, Snell T, Knapp M. Treatment paths and costs for young adults with acquired brain injury in the United Kingdom. Brain Inj. 2009;23(1):30-8.
8. Harrison D PG, Grieve R, Harvey S, Sadique Z. . Risk Adjustment In Neurocritical care (RAIN) - prospective validation of risk prediction models for adult patients with acute traumatic brain injury to use to evaluate the optimum location and comparative costs of neurocritical care: a cohort study. Health Technol Assess. 2013;17(23).
9. The CRASH-3 trial collaborators. Effects of tranexamic acid on death, disability, vascular occlusive events and other morbidities in patients with acute traumatic brain injury (CRASH-3): a randomised, placebo-controlled trial. Lancet. 2019;394:1713-23.
10. NHS. National schedule of reference costs 2017-18. 2018.
11. World Health Organisation. WHO CHOICE: Pakistan [Available from: https://www.who.int/choice/country/pak/cost/en/].
